# Interparietal Hernia Associated with Recurrent Inguinal Hernia: A Case Report

**DOI:** 10.70352/scrj.cr.26-0391

**Published:** 2026-07-16

**Authors:** Takahiro Saito, Koji Hontani, Takashi Kuroda, Sato Nishida, Tomoya Matsuda, Taku Maejima, Susumu Fukahori, Seiichi Minami, Masahiro Hagiwara, Satoshi Hirano

**Affiliations:** 1 Department of Surgery, Sapporo Higashi Tokusyukai Hospital, Sapporo, Hokkaido, Japan; 2 Department of Gastroenterological Surgery II, Hokkaido University Faculty of Medicine, Sapporo, Hokkaido, Japan

**Keywords:** interparietal hernia, interstitial hernia, recurrent inguinal hernia, transabdominal preperitoneal repair, CT, laparoscopic repair

## Abstract

**INTRODUCTION:**

Interparietal hernia (IPH) is a rare subtype of inguinal hernia in which the hernial sac extends between the layers of the abdominal wall musculature. Due to its unusual anatomical location, preoperative diagnosis is often difficult. Reports of adult cases in the English-language literature remain extremely limited. IPH occurring in association with a recurrent inguinal hernia is even rarer.

**CASE PRESENTATION:**

A man in his 70s with a history of bilateral inguinal hernia repair presented with abdominal pain and constipation. Contrast-enhanced CT revealed herniation of the sigmoid colon from the internal inguinal ring, with the hernial sac extending cranially between the external and internal oblique muscles. Based on these findings, an interstitial IPH associated with a recurrent inguinal hernia was suspected. Manual reduction was successfully performed, and elective surgery was planned. Subsequently, laparoscopic transabdominal preperitoneal repair (TAPP) was performed. Intraoperative findings confirmed a recurrent inguinal hernia with cranial extension of the hernia sac between the abdominal wall muscle layers. After adequate dissection of the preperitoneal space, a 17 × 12-cm self-gripping mesh (ProGrip) was placed to cover the entire myopectineal orifice. The postoperative course was uneventful, and the patient was discharged on POD 2. No recurrence was observed during the 6-month follow-up period.

**CONCLUSIONS:**

IPH associated with recurrent inguinal hernia is extremely rare. Careful interpretation of preoperative CT findings may facilitate the accurate recognition of this unusual hernia subtype and assist surgeons in selecting an appropriate surgical strategy. Awareness of this rare condition may improve preoperative assessment and operative planning in patients with atypical inguinal hernias.

## Abbreviations

IPH
: interparietal hernia
TAPP
: transabdominal preperitoneal repair

## INTRODUCTION

IPH is a rare subtype of inguinal hernia in which the hernial sac extends between the layers of the abdominal wall musculature rather than descending into the inguinal canal or scrotum.^1)^ IPH is classified into 3 types based on the location of the hernia sac (**Fig. 1**): preperitoneal, interstitial, and superficial. Among these, the interstitial type, characterized by the extension of the hernial sac between the abdominal wall muscle layers, is the most common.

**Fig. 1:**
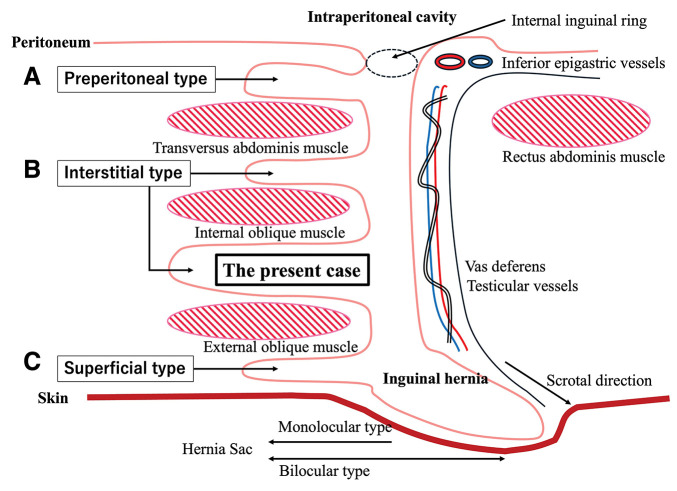
Schematic illustration of IPH. IPH is classified into 3 types according to the anatomical location of the hernial sac: (**A**) preperitoneal, (**B**) interstitial, and (**C**) superficial. The present case corresponds to the interstitial type, with the hernial sac extending between the external oblique and internal oblique muscles. This classification facilitates understanding of the anatomical plane of hernial sac extension in the present case.

Due to its unusual anatomical location, IPH may present with atypical clinical findings and can be difficult to diagnose preoperatively. Although numerous cases have been reported in the Japanese literature, only selected representative reports are cited here.^2–4)^ Adult cases reported in the English-language literature remain extremely limited.^5–11)^ We report a rare case of interstitial IPH associated with recurrent inguinal hernia that was successfully repaired by TAPP.

## CASE PRESENTATION

A man in his 70s presented to the gastroenterology outpatient clinic with abdominal pain and constipation. Contrast-enhanced CT was performed to investigate the cause of abdominal pain, revealing herniation of the sigmoid colon through the left internal inguinal ring. The patient was subsequently referred to the surgical department.

The patient had undergone right inguinal hernia repair 37 years previously and bilateral inguinal hernia repair 28 years previously. The operative details are unknown, although the patient reported that the mesh had been used in a previous surgery.

Physical examination revealed a bulge in the left lower abdominal wall (**Fig. 2**). The bulge was located cranial to the inguinal ligament and extended laterally. Mild tension was palpable within the bulge. No extension toward the scrotum was observed. Surgical scars were present in both inguinal regions, consistent with previous repairs via an anterior approach. Contrast-enhanced CT revealed herniation of the sigmoid colon from the internal inguinal ring toward the abdominal wall. No bowel wall thickening, mesenteric congestion, or other findings suggestive of strangulation were observed. The hernial sac extended cranially between the external and internal oblique muscles, suggesting an IPH (**Fig. 3**). The hernial sac did not extend into the scrotum and appeared monolocular. Based on these findings, a recurrent inguinal hernia associated with an IPH was suspected. Although a subtle direct recurrence may be recognizable retrospectively on CT, it was not identified preoperatively.

**Fig. 2:**
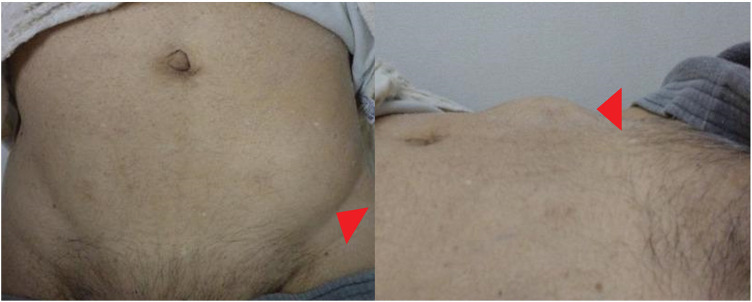
Physical findings. A bulge is observed in the left lower abdomen on physical examination (red triangle).

**Fig. 3:**
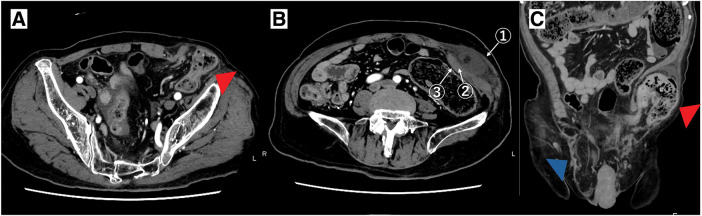
Preoperative CT findings. (**A**) Axial CT image showing the hernia sac protruding from the left internal inguinal ring (red triangle). (**B**) Axial CT image demonstrating the hernial sac extending between the external oblique and internal oblique muscles, consistent with an interstitial-type IPH. ➀ External oblique muscle; ➁ internal oblique muscle; ➂ transversus abdominis muscle. (**C**) Coronal CT image demonstrating the overall extent of the left IPH (red triangle), extending cranially and laterally within the abdominal wall. A recurrent right inguinal hernia is also observed. Near the recurrent site, findings suggest that previously placed mesh (blue triangle) is located in the subcutaneous layer (onlay position), rather than in the preperitoneal space, suggesting a prior anterior repair. IPH, interparietal hernia

Manual reduction of the hernia was successfully performed (**Fig. 4**), and elective surgery was scheduled. Because previous surgeries were presumed to have been performed via an anterior approach, laparoscopic surgery was selected to evaluate the recurrent hernia and preperitoneal space from the intraperitoneal side.

**Fig. 4:**
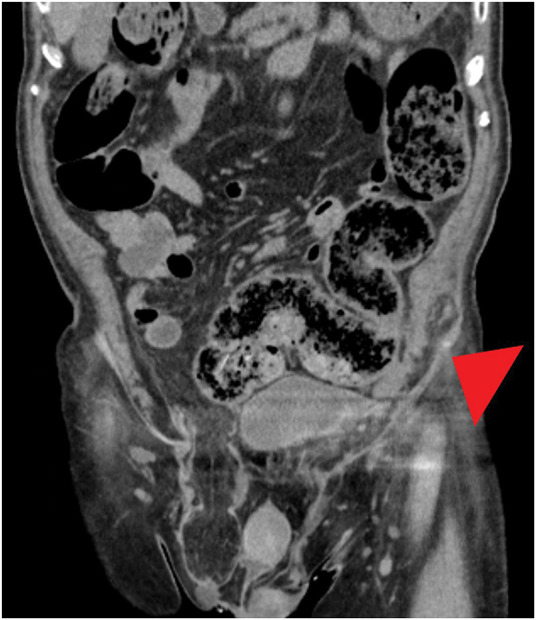
CT findings after manual reduction. Follow-up CT performed after successful manual reduction demonstrates disappearance of the herniated sigmoid colon (red triangle).

A 12-mm port was placed at the umbilicus, and two 5-mm ports were inserted bilaterally in the lower abdomen. Laparoscopic observation revealed bilateral recurrent inguinal hernias. According to the Japanese Hernia Society classification, the left lesion was classified as R1M1 and Unclassified, reflecting the coexistence of a direct recurrent hernia and an IPH categorized as an Unclassified type (**Fig. 5A**). The right lesion was classified as R2M1 (**Fig. 5B**). Because a sliding hernia involving the sigmoid colon was observed on the left side, an additional 5-mm port was inserted to assist retraction. The peritoneum was incised circumferentially around the hernia orifice, the hernial sac was carefully dissected, and the preperitoneal space was developed. This approach was selected because the hernial sac extended cranially toward the abdominal wall, making traction and dissection of the hernia sac technically challenging. Although most dissections proceeded without difficulty, fibrotic changes were observed on the cranial side of the hernia orifice, making peritoneal dissection slightly more challenging in that area. No mesh from a previous surgery was identified in the preperitoneal space. After adequate dissection (**Fig. 5C**), a self-gripping mesh (ProGrip; Medtronic, Dublin, Ireland), trimmed to 17 × 12 cm, was placed to cover the entire myopectineal orifice (**Fig. 5D**), and the peritoneum was then closed. The right side was repaired in the same manner using a mesh trimmed to 14 × 9 cm.

**Fig. 5:**
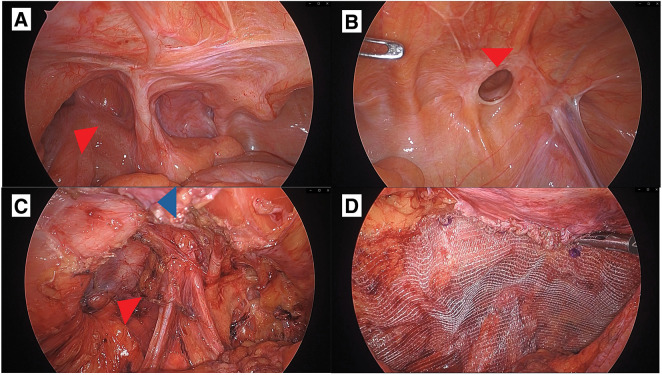
Intraoperative findings during laparoscopic repair. (**A**) Intraoperative view of the left side demonstrating a recurrent direct hernia (M1) and an IPH extending cranially between the external oblique and internal oblique muscles (red triangle), corresponding to an Unclassified type in the Japanese Hernia Society classification. (**B**) Intraoperative view of the right side before dissection (red triangle), demonstrating a recurrent direct hernia (R2M1). (**C**) The peritoneum is incised circumferentially around the hernia orifice, and the hernial sac is carefully dissected (red triangle). Dense fibrotic changes are observed in the cranial peritoneum (blue triangle), making peritoneal dissection more challenging in this area. (**D**) After adequate dissection of the preperitoneal space, a 17 × 12-cm self-gripping mesh (ProGrip) was placed to cover the entire myopectineal orifice.

The postoperative course was uneventful, and the patient was discharged on POD 2. The patient remained asymptomatic with no evidence of recurrence during a 6-month follow-up period.

## DISCUSSION

The present case demonstrates a rare form of recurrent inguinal hernia presenting as an IPH and highlights the importance of preoperative imaging and an appropriate surgical strategy. IPH is a rare subtype of inguinal hernia in which the hernial sac extends between the layers of the abdominal wall rather than progressing along the inguinal canal toward the scrotum.^1)^ Because the hernial sac spreads within the abdominal wall layers, the typical scrotal swelling observed in ordinary inguinal hernias may be absent, making clinical diagnosis difficult.

Based on the anatomical location of the hernial sac, IPHs are classified into preperitoneal, interstitial, and superficial types (**Fig. 1**). Among these, the interstitial type, characterized by extension of the hernial sac between the abdominal wall muscle layers, is the most frequently reported subtype. According to the Japanese Hernia Society classification, IPH is categorized as an Unclassified type rather than a lateral hernia,^12)^ even when it originates from the internal inguinal ring, because the hernial sac does not traverse the inguinal canal. Owing to this unusual herniation pathway, IPH may present with atypical clinical findings and may not be readily recognized before surgery.

Adult cases of IPH associated with inguinal hernia are rare. A review of the English-language literature identified 8 previously reported adult cases, the clinical characteristics of which are summarized in **Table 1**.^5–11)^ Most reported cases were classified as the interstitial type and were successfully treated surgically. Although preoperative diagnosis of IPH can be challenging, several reported cases, including the present case, demonstrate the usefulness of CT for diagnosis and surgical planning. Read and Gilbert reported 3 cases of interstitial recurrence after Lichtenstein repair, one of which clearly demonstrated extension of the hernial sac within the abdominal wall and was therefore included in our review.^5)^ In the remaining 2 cases, recurrent indirect hernias arising from the internal inguinal ring beneath the prosthetic mesh were described; however, the anatomical relationship between the recurrent sac and the abdominal wall muscle layers was not clearly documented. Therefore, classification of these cases as IPHs remains uncertain. In the present case, preoperative CT clearly demonstrated cranial extension of the hernial sac between the abdominal wall muscle layers. Notably, this region corresponded to the area where dense fibrotic changes were encountered intraoperatively.

**Table 1:** Summary of previously reported adult cases of IPH associated with inguinal hernia

Author	Year	Age/sex	Side	Physical exam	Prior repair	Preop imaging/diagnosis	IPH subtype	Repair
Read and Gilbert^5)^	2004	47/M	L	Mass at the linea semilunaris	Anterior repair	None/inguinal hernia	Interstitial/monolocular	Anterior approach
Glaser et al.^6)^	2011	68/F	R	Mass cephalad to the inguinal ligament	None	CT/inguinal hernia	Interstitial/monolocular	TEP
Sakamoto et al.^7)^	2016	51/M	R	Pain only, no significant findings	None	CT/IPH or internal hernia	Preperitoneal/monolocular	TAPP
Kumar et al.^8)^	2018	62/M	R	Large iliac fossa mass	None	CT/spigelian hernia	Interstitial/monolocular	Anterior approach
Cervantes et al.^9)^	2020	85/F	R	Large umbilical and inguinal mass	None	CT/spigelian hernia	Interstitial/monolocular	Anterior approach
Ozawa et al.^10)^	2022	79/M	R	Inguinal swelling toward the head	None	CT/IPH	Interstitial/monolocular	TAPP
Ozawa et al.^10)^	2022	67/M	R	Inguinal swelling toward the head	None	CT/IPH	Interstitial/monolocular	TAPP
Althawadi et al.^11)^	2024	44/M	R	Rebound tenderness in the iliac fossa	None	CT/ventral hernia	Interstitial/monolocular	TAPP
Our case	2026	76/M	L	Bulge cranial to the inguinal ligament	Anterior repair	CT/IPH	Interstitial/monolocular	TAPP

F, female; IPH, interparietal hernia; L, left; M, male; Preop, preoperative; R, right; TAPP, transabdominal preperitoneal repair; TEP, totally extraperitoneal repair

Preoperative imaging plays an important role in the diagnosis of this rare condition. As the hernial sac extends between the layers of the abdominal wall rather than toward the scrotum, the clinical findings may be atypical and easily misinterpreted as a conventional inguinal hernia. In the present case, contrast-enhanced CT clearly demonstrated the cranial extension of the hernial sac between the external and internal oblique muscles, enabling an accurate preoperative diagnosis. Therefore, CT is particularly useful for identifying unusual hernial pathways and planning optimal surgical approaches. In the present case, the atypical bulge was located cranially to the usual inguinal region, making diagnosis based on physical examination alone difficult. Preoperative CT demonstrated that the lesion represented a recurrent inguinal hernia with cranial extension between the abdominal wall muscle layers. Recognition of this unusual anatomy facilitated operative planning, including careful dissection of the interparietal space and adequate mesh coverage during TAPP repair.

Although numerous cases have been reported in the Japanese literature, only selected representative reports are cited here.^2–4)^ Adult cases reported in the English-language literature remain extremely limited.^5–11)^ A plausible explanation for the relatively high number of reports in Japan is the frequent use of CT in clinical practice. International guidelines for groin hernia management,^13)^ as well as Japanese guidelines,^14)^ do not recommend routine preoperative imaging for typical inguinal hernia. However, in real-world clinical practice, CT is frequently performed on patients presenting with abdominal pain or suspected incarceration and is readily available in the Japanese healthcare system. As a result, unusual hernia subtypes, such as IPH, may be detected incidentally on imaging. Therefore, IPH may be under-recognized in many clinical settings.

Regarding surgical management, laparoscopic repair offers several advantages for unusual or recurrent hernias because the anatomy of the myopectineal orifice and hernia pathway can be directly evaluated from the intraperitoneal side. In the present case, TAPP repair was selected to allow a precise assessment of the anatomy of the recurrent hernia. Because the hernial sac extended cranially toward the abdominal wall, traction of the sac was difficult, and the peritoneum was incised circumferentially around the hernial orifice. This approach facilitates safe dissection of the preperitoneal space and adequate reduction of the hernial sac. After sufficient dissection, a self-gripping mesh was placed to cover the entire myopectineal orifice.

The mechanism underlying recurrent IPH after anterior inguinal hernia repair remains unclear. Read et al. reported 3 cases of interstitial recurrence following Lichtenstein repair and suggested that recurrent indirect herniation may result from an overlooked indirect sac or a persistent processus vaginalis at the time of the primary repair.^5)^ Although the details of the initial operation were unavailable in the present case, an occult indirect component or inadequate treatment of the hernia sac may have contributed to recurrence. The previously implanted mesh and postoperative scarring may also have altered the normal anatomical planes and influenced the direction of hernia sac progression. Rather than following the usual inguinal canal pathway, the recurrent hernia sac may have extended into the intermuscular plane along a path of lower resistance. Furthermore, insufficient reinforcement of the lateral triangle or postoperative mesh shrinkage may have facilitated cranio-lateral extension of the hernial sac from the internal inguinal ring. However, these proposed mechanisms do not fully explain the development of interparietal recurrence. If the recurrence had been caused solely by an overlooked indirect sac, it might have been expected to occur earlier than observed in the present case. Moreover, the above factors may also be present in ordinary recurrent inguinal hernias, yet recurrent IPH remains exceedingly rare. Therefore, additional factors, including anatomical changes related to the previous anterior repair, such as muscular injury or disruption of normal tissue planes, may also have contributed to this unusual recurrence pattern. However, because recurrent inguinal hernia is common whereas IPH remains extremely rare, the relationship between the 2 conditions remains speculative. The present case does not establish a causal association, and further accumulation of similar cases will be required to clarify the underlying mechanism.

## CONCLUSIONS

In conclusion, IPH associated with recurrent inguinal hernia is extremely rare. Preoperative CT is useful for recognizing this unusual hernia subtype and planning appropriate surgical management. TAPP repair allows detailed evaluation of the hernia anatomy and facilitates safe and effective treatment. Recognition of this rare subtype may assist surgeons in selecting the optimal operative strategy.

## DECLARATIONS

### Availability of data and materials

The datasets supporting the conclusions of this article are included within the article and its additional files.
